# Myo2p is the major motor involved in actomyosin ring contraction in fission yeast

**DOI:** 10.1016/j.cub.2016.12.024

**Published:** 2017-02-06

**Authors:** Paola Zambon, Saravanan Palani, Anton Kamnev, Mohan K. Balasubramanian

**Affiliations:** 1 Division of Biomedical Sciences, Warwick Medical School, University of Warwick, Coventry, CV4 7AL, UK

## Abstract

Cytokinesis in many eukaryotes requires an actomyosin-based contractile ring [1]. In fission yeast, cytokinesis involves the type II myosins Myo2p and Myp2p and the type V myosin Myo51p [2]. A recent study by Laplante *et al.*[3], using deletion mutants of *myp2* and *myo51* and the mis-sense mutant *myo2*-E1 [4], concluded that each myosin has distinct functions and proposed that Myp2p plays the dominant role in actomyosin ring contraction. Here we present evidence that Myo2p, not Myp2p, is likely to be the major motor driving actomyosin ring contractility. Since the previous work [3] was performed at 25°C, the permissive temperature for *myo2*-E1, we compared cytokinesis timings in *myo2*-E1 and *myo2*Δ at 25°C and found that *myo2*-E1 is only partially compromised at 25°C. Furthermore, we find that *myp2*Δ and *myp2*Δ *myo51*Δ double mutants contract actomyosin rings at ∼90% of the rate of wild-type cells at 30°C and 36°C, suggesting that Myp2p plays a minimal role in ring contraction at these temperatures. Finally, ring contraction in our *myo2*-E1 strain took longer at 25°C than previously reported [3]. Although faster-acting alleles of *myo2* will be required to evaluate its contribution at 25°C, our work establishes that Myo2p is the major motor involved in ring contraction, under most, if not all, conditions.

## Teaser

Three myosins participate in the cytokinetic actomyosin ring in fission yeast. Zambon et al. find that the type II myosin Myo2p is key to both assembly and contraction of the actomyosin ring, while the second myosin II isoform Myp2p is fully dispensable for actomyosin ring assembly and participates in its contraction only at lower temperatures.

## Main Text

**Figure 1:**
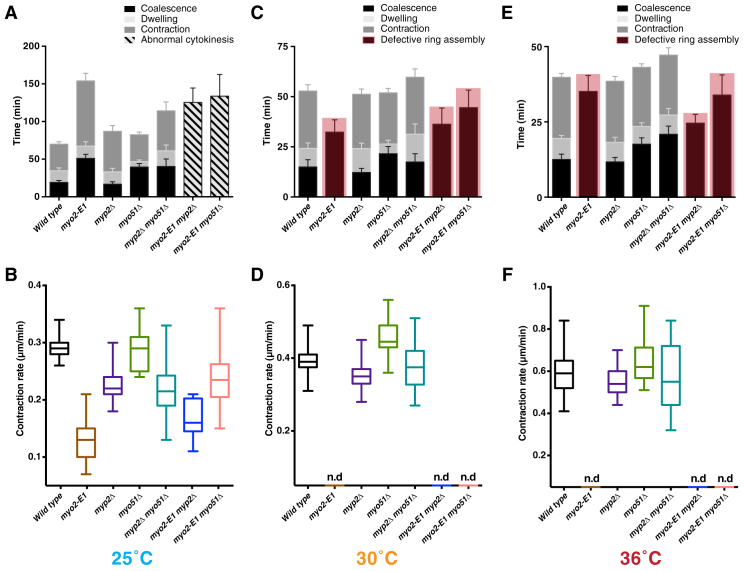
Actomyosin ring kinetics in S. pombe myosin mutants. (A,C,E) Time taken for various steps in cytokinesis (coalescence of nodes into a ring, dwell time before contraction, and contraction). (B,D,F) Ring contraction rates. In all cases, Rlc1p-3GFP was used as a marker of cytokinetic nodes and the actomyosin ring. Note that improper rings that underwent aberrant contraction were detected in *myo2*-E1 mutants (at 30°C and 36°C), *myo2*-E1 *myp2*Δ mutants (at 25°C, 30°C and 36°C) and *myo2*-E1 *myo51*Δ mutants (at 25°C, 30°C and 36°C).

Work from several laboratories has shown that *myo2*-E1 forms healthy colonies at 25°C but fails to do so at increased temperatures 4, 5. Since previous work has shown that Myo2-E1p (the product of *myo2*-E1) neither binds actin filaments nor has ATPase activity *in vitro* at 25°C [6], Laplante *et al.* [3] inferred that *myo2*-E1 should also be severely compromised *in vivo* at 25°C. It is known that *in vitro* activity and *in vivo* function of products of mutant alleles are not necessarily correlated [7]. Furthermore, Laplante *et al.* [3] extrapolate that, since *myo2*-E1 is a severely compromised allele of *myo2*, the mGFP-tagged version (mGFP-*myo2*-E1) used in their work would also be severely compromised. The data in Figure 1D of the accompanying response of Laplante and Pollard and in Figure S1A in Laplante *et al.* [3] suggest, however, that the mGFP tag partially rescues *myo2*-E1.

We first investigated whether *myo2*-E1 is an appropriate allele to understand Myo2p function at 25°C. We imaged the dynamics of the actomyosin ring component Blt1p, which localizes to precursor nodes and actomyosin rings [8], in germinating *myo2*Δ spores, with the rationale that for its use in comparative studies it should have a phenotype comparable to *myo2*Δ. Actomyosin rings never assembled in *myo2*Δ cells, even after 4 hours of imaging, whereas germinated wild-type spores assembled normal actomyosin rings (Figure S1A and Movie S1 in Supplemental Information). However, contractile actomyosin rings assembled in *myo2*-E1 cells and importantly even in *myo2*-E1 *myo51*Δ cells [3] (Figure S1B, see 36’–54’ time points, and Movie S2, see 35’–55’ time points), suggesting that Myo2-E1p should retain some activity for ring assembly through the search, capture, pull and release (SCPR) mechanism [9]. Thus, *myo2*-E1 is not a severely compromised allele at 25°C and is inappropriate for investigating the contribution of Myo2p at 25°C.

We then compared the time taken for various aspects of cytokinesis in wild-type cells, the partially active *myo2*-E1 mutant, the *myp2*Δ *myo51*Δ double mutant, and double mutants combining the partially compromised *myo2*-E1 and *myp2*Δ or *myo51*Δ at 25°C (as a control), and at 30°C and 36°C. Through DNA sequencing and genetic crosses, we ensured that all the *myo2*-E1 strains used only carried the previously described G345R mutation and did not carry second-site modifiers.

At 25°C, we made similar observations to Laplante *et al.* [3] when ring assembly, maturation, and contraction times were scored in wild-type cells, and in *myp2*Δ, and *myo51*Δ mutants, but not in *myo2*-E1 and *myp2*Δ *myo51*Δ mutants (Figures 1A and S1B, and Movie S2). Ring assembly time doubled in *myo51*Δ compared with wild-type cells, consistent with previous work reporting an ancillary role for Myo51p in ring assembly [10]. Ring contraction was slower in *myp2*Δ mutants (53.9 ± 7.5 minutes). In our analysis, ring contraction was not as severely affected in *myp2*Δ *myo51*Δ as reported in Laplante *et al.* [3]. Importantly, even the partially compromised *myo2*-E1 (containing Myp2p and Myo51p) had a stronger defect than *myp2*Δ in ring contraction and took ∼86 mins for ring contraction, suggesting that Myo2p rather than Myp2p played a major role in ring contraction even at 25°C, with Myp2p playing an ancillary role in ring contraction at 25°C (Figures 1A,B and S1B and Movie S2). The strong additive effect in *myo2*-E1 *myo51*Δ and *myo2*-E1 *myp2*Δ mutants made it difficult to demarcate different steps in cytokinesis, but the entire process of improper cytokinesis took a comparable amount of time in *myo2*-E1, *myo2*-E1 *myp2*Δ, and *myo2*-E1 *myo51*Δ mutants (125–150 minutes).

Ring assembly was defective in *myo2*-E1, *myo2*-E1 *myo51*Δ, and *myo2*-E1 *myp2*Δ mutants grown at 30°C or 36°C, confirming the essential role of Myo2p in actomyosin ring assembly (Figures 1C,E and S1C,D and Movie S2). Ring contraction times in *myp2*Δ, *myo51*Δ, and *myp2*Δ *myo51*Δ mutants grown at 30°C or 36°C were comparable to those in wild-type cells, with ring contraction rates at least 90% of that seen in wild-type cells (Figures 1D,F and S1C,D and Movie S2). These data further reinforced the view that Myp2p is not the major motor involved in ring contraction at 30°C and 36°C.

Laplante and Pollard (accompanying response) have shown that Rlc1p-3GFP shows adverse interactions specifically with *myo2*-E1 and suggest this may have increased the ring contraction time in *myo2*-E1 *rlc1*-3GFP at 25°C in our work. However, the fact that *rlc1*-3GFP exacerbates the cytokinesis defect of the *myo2*-E1 mutant (but not wild-type cells) is itself further evidence that *myo2*-E1 is a weak allele that is further weakened by its interaction with Rlc1p-3GFP *in vivo*. This further weakened Myo2-E1p–Rlc1p-3GFP complex causes a significant reduction in ring contraction rate, strengthening our conclusion that Myo2p is the main motor involved in ring contraction.

What then are the specialized functions of Myo2p, Myp2p, and Myo51p? We and others have established that Myo2p is central to ring assembly at all temperatures (current work and [2]), with Myo51p playing an ancillary role [10]. Our analysis has established that ring contraction is independent of Myp2p at 30°C or 36°C. Myp2p may perform an ancillary role in ring contraction at these temperatures. As reported [3], ring contraction is slower in cells lacking Myp2p at 25°C, consistent with previous work that Myp2p is specialized for cytokinesis at lower temperatures [2]. In our work, actomyosin ring contraction in the *myo2*-E1 mutant took longer than in the *myp2*Δ mutant at 25°C, suggesting that Myo2p is likely the major motor involved in ring contraction even at 25°C. However, analysis of *myo2*-E1 mutants to inform the role of Myo2p in ring contraction should be considered to underestimate Myo2p’s function, since *myo2*Δ cells and *myo2*-E1 *rlc1*-3GFP cells have a stronger phenotype than *myo2*-E1 at 25°C. Fast-acting stronger conditional mutant alleles of Myo2p will be essential to investigate the precise role of Myo2p in ring contraction at 25°C.

## Author contributions

P.Z. and S.P., conception and design, acquisition of data, analysis and interpretation of data, drafting or revising the article; A.K., analysis tool development and interpretation of data; M.K.B., conceived the project, conception and design, analysis and interpretation of data; S.P. and M.K.B., wrote the manuscript; all authors reviewed the manuscript.

## Supplemental Information

Document S1. Experimental Procedures and One Figure

Movie S1. Actomyosin ring assembly and contraction of wild-type and *myo2Δ* spore Time lapse movies of mitotic cells of wild-type Blt1-GFP and *myo2*Δ Blt1-GFP germinated from spores. Time-lapse images were acquired by spinning disk microscopy (Andor Revolution XD imaging system) at 25˚C. Cells were imaged using YES agarose pad imaging method. Blt1-GFP (membrane bound ring anchoring protein) served as contractile ring marker. Scale bar represent 3μm.

Movie S2. Actomyosin ring assembly and contraction of wild-type, *myp2*Δ, *myo51*Δ, *myp2*Δ *myo51*Δ, *myo2*-E1, *myo2*-E1 *myp2*Δ, *myo2*-E1 *myo51*Δ Time lapse movies of mitotic cells of 7 genotypes (wild-type, *myo2*-E1, *myp2*Δ, *myo51*Δ, *myp2*Δ *myo51*Δ, *myo2*-E1 *myp2*Δ, *myo2*-E1 *myo51*Δ) respectively. Time-lapse movies were acquired by spinning disk microscopy (Andor Revolution XD imaging system) at different temperatures (25˚C, 30˚C and 36˚C). Cells were imaged using YES agarose pad imaging method. Rlc1-3GFP (myosin regulatory light chain 1), which served as contractile ring marker and Alpha tubulin2 (mCherry-atb2) served as a cell cycle marker (t=0 denotes the elongation of the spindle ∼1 μm). Scale bar represent 3μm.
