# Supplementary material for: Myo2p is the major motor involved in actomyosin ring contraction in fission yeast
Source: Curr Biol. 2017 Feb 6;27(3):R99–R100. doi: 10.1016/j.cub.2016.12.024 (PMC5300900; doi:10.1016/j.cub.2016.12.024)
Supplement: Document S1. Experimental Procedures and One Figure [file mmc1.pdf]

Supplemental Information

Myo2p is the major motor involved in actomyosin ring contraction in fission yeast

Paola Zambon, Saravanan Palani, Anton Kamnev and Mohan K. Balasubramanian

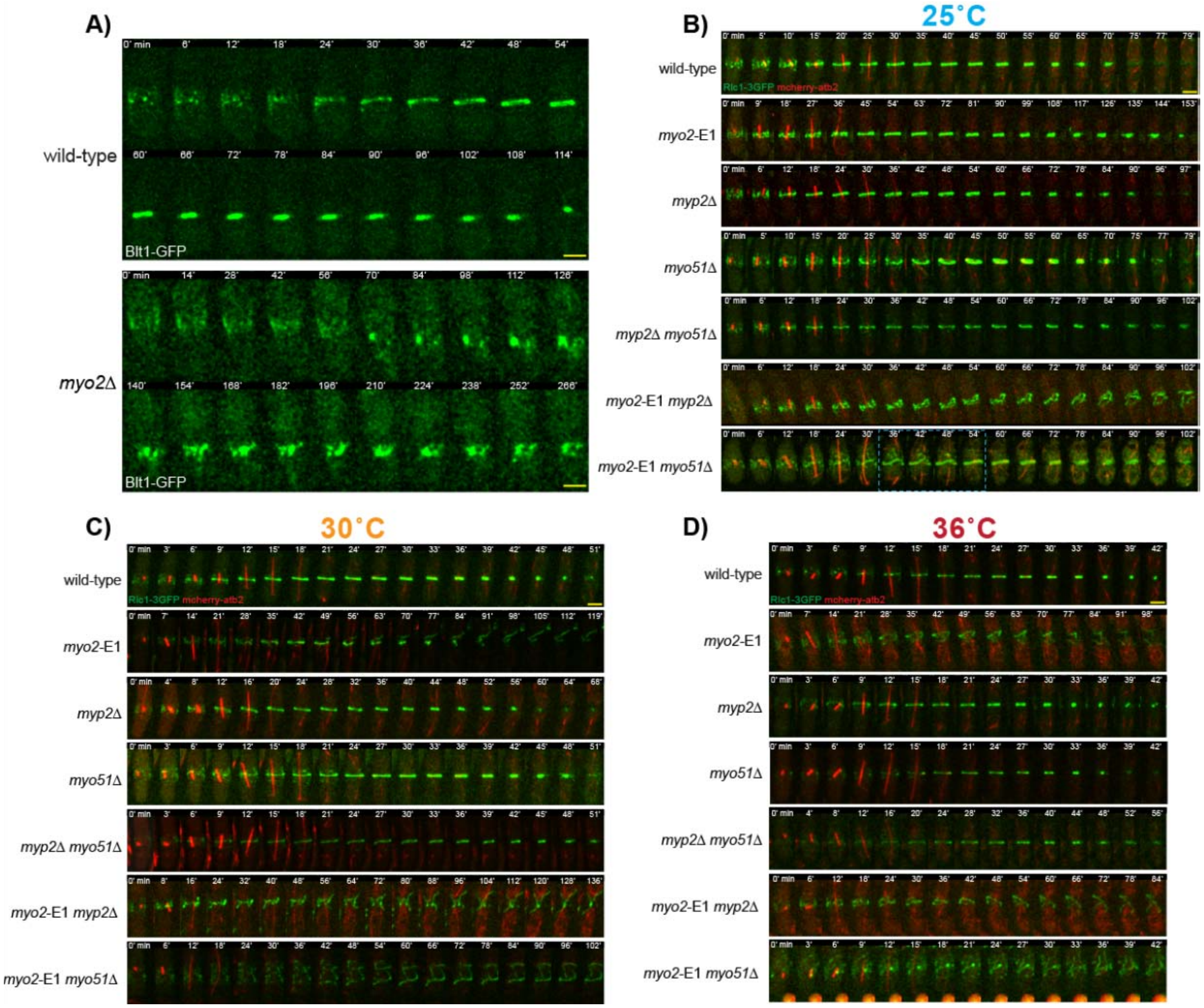

A)
wild-type
myo2Δ
Blt1-GFP
B)
25˚C
wild-type
myo2-E1
myp2Δ
myo51Δ
myp2Δ myo51Δ
myo2-E1 myp2Δ
myo2-E1 myo51Δ
Rlc1-3GFP mcherry-atb2
C)
30˚C
wild-type
myo2-E1
myp2Δ
myo51Δ
myp2Δ myo51Δ
myo2-E1 myp2Δ
myo2-E1 myo51Δ
Rlc1-3GFP mcherry-atb2
D)
36˚C
wild-type
myo2-E1
myp2Δ
myo51Δ
myp2Δ myo51Δ
myo2-E1 myp2Δ
myo2-E1 myo51Δ
Rlc1-3GFP mcherry-atb2

Figure S1. Actomyosin ring assembly, dwelling and contraction of wild type *S. pombe* and myosin mutants.

(A) Time-lapse image series of mitotic cells of wild-type Blt1-GFP and *myo2*Δ Blt1-GFP germinated from spores. Scale bars represent 3 μm. (B–D) Time-lapse image series of mitotic cells of seven genotypes (wild-type, *myo2*-E1, *myp2*Δ, *myo51*Δ, *myp2*Δ *myo51*Δ, *myo2*-E1 *myp2*Δ, *myo2*-E1 *myo51*Δ), respectively. In all cases, Rlc1-3GFP was used as a marker of cytokinetic nodes and the actomyosin ring and alpha tubulin 2 (mCherry-atb2) served as a cell-cycle marker (t = 0 denotes the elongation of the spindle ~1 μm). Time-lapse movies were taken at (B) 25˚C, (C) 30˚C and (D) 36˚C, respectively. In (B), time points between 33 and 54 minutes were highlighted with blue dotted square box in the *myo2*-E1 *myo51*Δ cells. Scale bars represent 3 μm.

Supplemental Experimental Procedures

Yeast strains and growth conditions

The *S. pombe* strains used in this study are listed below and all of them have been verified by PCR and DNA sequencing using appropriate primers. *S. pombe* cell culture, genetic and growth conditions for live-cell imaging were used as previously described [S1,S2].

Table of strains used in this study

MBY8841	mCherry-atb2::hph; Rlc1-3GFP::KanMX6; ura4-D18 ade6-210 leu1-32 h+
MBY10024-1	*myo2*-E1 mCherry-atb2::hph Rlc1-3GFP::KanMX6 h+
MBY10075	*myp2*::NatMX6 mCherry-atb2::hph; Rlc1-3GFP::KanMX6 ade6-21
MBY10097	*myo2*-E1 *myp2*::NatMX6 mCherry-atb2::hph Rlc1-3GFP::KanMX6 ade6-21
MBY10995	*myo51*::ura4 *myp2*::NatMX6 mCherry-atb2::hph Rlc1-3GFP::KanMX6
MBY10996	*myo51*::ura4 mCherry-atb2::hph Rlc1-3GFP::KanMX6
MBY11002	h90/h90 *myo2*Δ/+ strain a (*myo2*Δ::ura4) Blt1-GFP::NatMX6
MBY11129	*myo2*-E1 *myo51*::ura4 Rlc1-3GFP::KanMX6 mCherry-atb2::hph h+

Live-cell imaging

For time-lapse live-cell imaging, mid log phase cells were grown at 25°C and shifted to 30°C and/or 36°C for 3–4 hours prior to imaging. Time-lapse movies were taken under fully controlled 30°C and/or 36°C incubation chamber while the images were acquired for 3–4 hours. YES Agarose pad imaging method was used for time-lapse imaging as described [S2]. Time-lapse series were acquired using a spinning disk confocal microscope (Andor Revolution XD imaging system, equipped with a 100x oil immersion 1.45NA Nikon Plan Apo lambda, and a confocal unit Yokogawa CSU-X1, EMCCD detector (Andor iXON) and Andor iQ acquisition software. Fifteen z-stacks of 0.5 μm thicknesses at 1-minute intervals were taken for Rlc1-3GFP (myosin regulatory light chain 1), which served as contractile ring marker and alpha tubulin 2 (mCherry-atb2) served as a cell-cycle marker. We defined the timing of assembly from coalescence of nodes into a condensed single ring (t = 0 denotes the

elongation of the spindle ~1 μm). The time between full ring assembly and initiation of contraction was considered as dwelling time. We determined the timing of ring contraction from a complete ring into a dot or no fluorescence. Scale bar 3 μm. Images were processed using Fiji software.

Ring contraction rate measurement

The rate of ring contraction was measured similarly as described [S3]. First, kymographs of contracting cytokinetic ring (15 z-stacks of 0.5 μm thickness taken for 7 μm at 1-minute intervals) were constructed from maximum intensity projection of original time series along the z-axis. Next, ring contraction velocity was measured as a slope formed by migrating ring edge to the time-axis. On average we measured 20~30 rings per group.

Supplemental References

S1. Moreno, S., Klar, A., and Nurse, P. (1991). Molecular genetic analysis of fission yeast Schizosaccharomyces pombe. Methods Enzymol. *194*, 795-823.

S2. Huang, J., Huang, Y., Yu, H., Subramanian, D., Padmanabhan, A., Thadani, R., Tao, Y., Tang, X., Wedlich-Soldner, R., and Balasubramanian, M.K. (2012). Nonmedially assembled F-actin cables incorporate into the actomyosin ring in fission yeast. J. Cell Biol. *199*, 831-847.

S3. Laplante, C., Berro, J., Karatekin, E., Hernandez-Leyva, A., Lee, R., and Pollard, T.D. (2015). Three myosins contribute uniquely to the assembly and constriction of the fission yeast cytokinetic contractile ring. Curr. Biol. *25*, 1955-1965.
