# Supplementary figures and images for: Myo2p is the major motor involved in actomyosin ring contraction in fission yeast
Source: Curr Biol. 2017 Feb 6;27(3):R99–R100. doi: 10.1016/j.cub.2016.12.024 (PMC5300900; doi:10.1016/j.cub.2016.12.024)

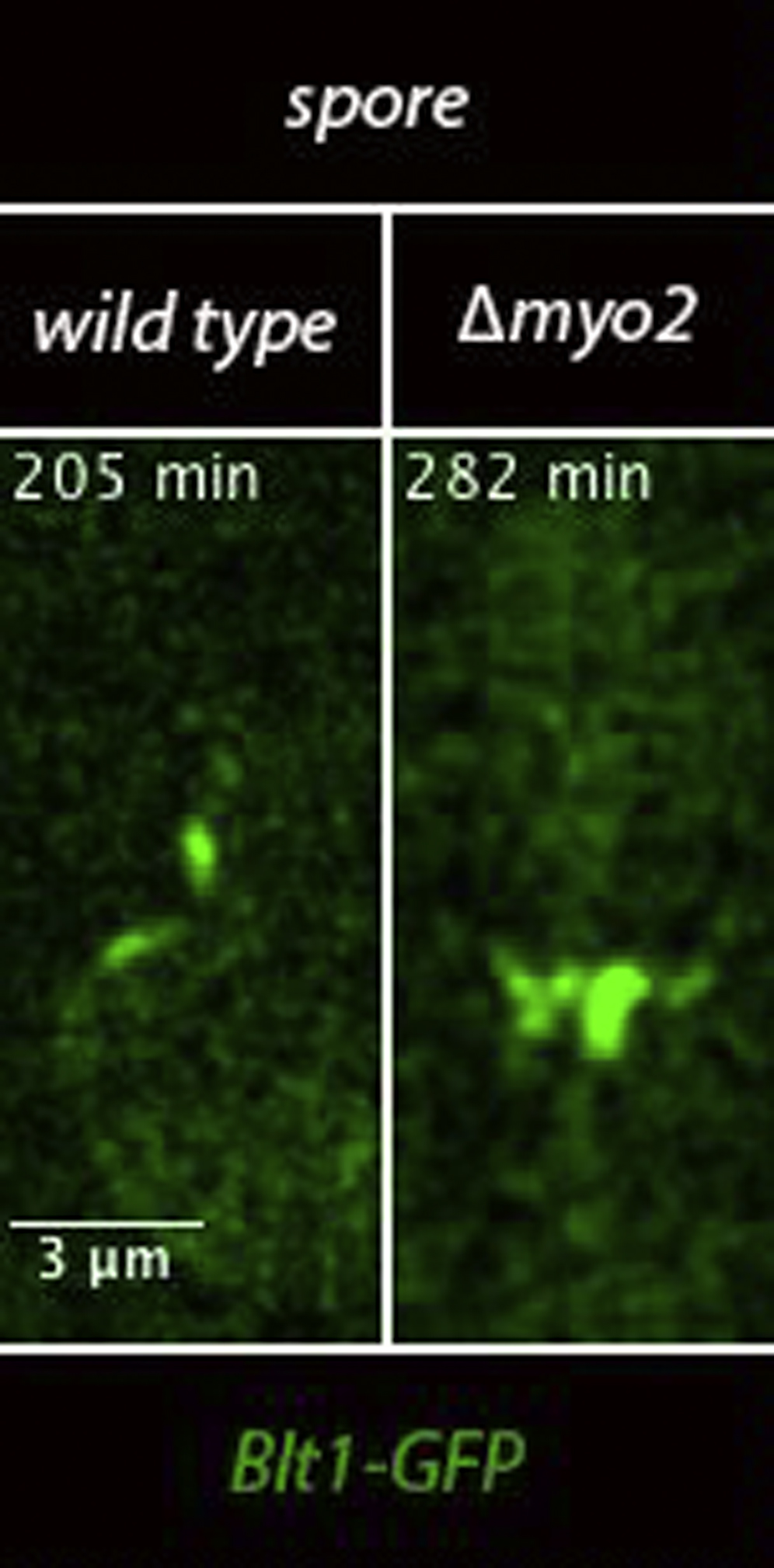

Supplement: Movie S1. Actomyosin ring assembly and contraction of wild-type and myo2Δ spore — Time lapse movies of mitotic cells of wild-type Blt1-GFP and myo2Δ Blt1-GFP germinated from spores. Time-lapse images were acquired by spinning disk microscopy (Andor Revolution XD imaging system) at 25˚C. Cells were imaged using YES agarose pad imaging method. Blt1-GFP (membrane bound ring anchoring protein) served as contractile ring marker. Scale bar represent 3μm. [file mmc2.jpg]

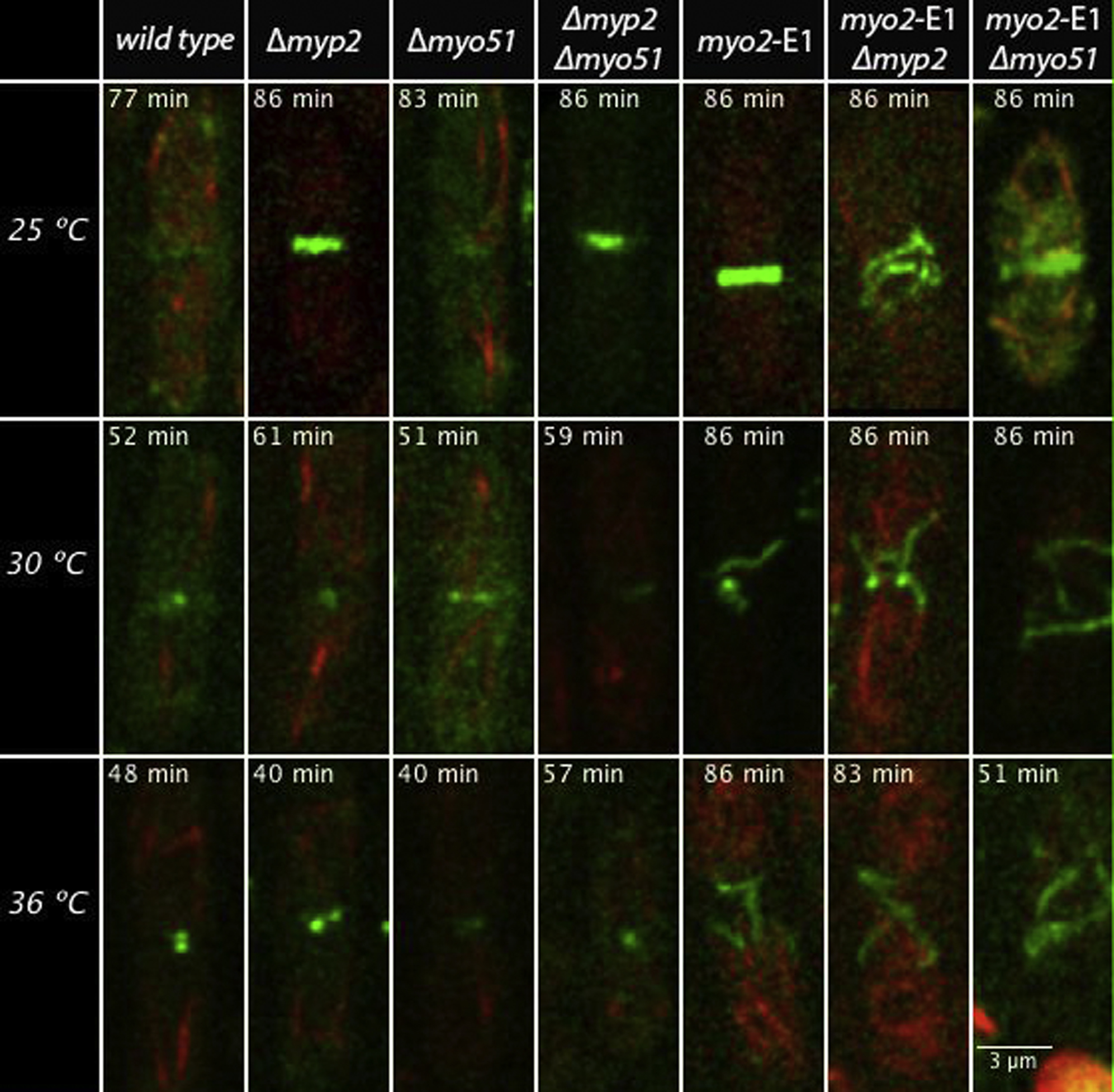

Supplement: Movie S2. Actomyosin ring assembly and contraction of wild-type, myp2Δ, myo51Δ, myp2Δ myo51Δ, myo2-E1, myo2-E1 myp2Δ, myo2-E1 myo51Δ — Time lapse movies of mitotic cells of 7 genotypes (wild-type, myo2-E1, myp2Δ, myo51Δ, myp2Δ myo51Δ, myo2-E1 myp2Δ, myo2-E1 myo51Δ) respectively. Time-lapse movies were acquired by spinning disk microscopy (Andor Revolution XD imaging system) at different temperatures (25˚C, 30˚C and 36˚C). Cells were imaged using YES agarose pad imaging method. Rlc1-3GFP (myosin regulatory light chain 1), which served as contractile ring marker and Alpha tubulin2 (mCherry-atb2) served as a cell cycle marker (t=0 denotes the elongation of the spindle ∼1 μm). Scale bar represent 3μm. [file mmc3.jpg]
